# Precision gynecologic oncology: circulating cell free DNA epigenomic analysis, artificial intelligence and the accurate detection of ovarian cancer

**DOI:** 10.1038/s41598-022-23149-1

**Published:** 2022-11-03

**Authors:** Ray O. Bahado-Singh, Amin Ibrahim, Zaid Al-Wahab, Buket Aydas, Uppala Radhakrishna, Ali Yilmaz, Sangeetha Vishweswaraiah

**Affiliations:** 1 Department of Obstetrics and Gynecology, Oakland University-William Beaumont School of Medicine, Royal Oak, MI USA; 2 Department of Obstetrics and Gynecology, Beaumont Research Institute, 3811 W. 13 Mile Road, Royal Oak, MI 48073 USA; 3 Department of Care Management Analytics, Blue Cross Blue Shield of Michigan, Detroit, MI USA

**Keywords:** Tumour biomarkers, Gynaecological cancer, Ovarian cancer, Cancer, Cancer genomics

## Abstract

Ovarian cancer (OC) is the most lethal gynecologic cancer due primarily to its asymptomatic nature and late stage at diagnosis. The development of non-invasive markers is an urgent priority. We report the accurate detection of epithelial OC using Artificial Intelligence (AI) and genome-wide epigenetic analysis of circulating cell free tumor DNA (cfTDNA). In a prospective study, we performed genome-wide DNA methylation profiling of cytosine (CpG) markers. Both conventional logistic regression and six AI platforms were used for OC detection. Further, we performed Gene Set Enrichment Analysis (GSEA) analysis to elucidate the molecular pathogenesis of OC. A total of 179,238 CpGs were significantly differentially methylated (FDR p-value < 0.05) genome-wide in OC. High OC diagnostic accuracies were achieved. Conventional logistic regression achieved an area under the ROC curve (AUC) [95% CI] 0.99 [± 0.1] with 95% sensitivity and 100% specificity. Multiple AI platforms each achieved high diagnostic accuracies (AUC = 0.99–1.00). For example, for Deep Learning (DL)/AI AUC = 1.00, sensitivity = 100% and 88% specificity. In terms of OC pathogenesis: GSEA analysis identified ‘Adipogenesis’ and ‘retinoblastoma gene in cancer’ as the top perturbed molecular pathway in OC. This finding of epigenomic dysregulation of molecular pathways that have been previously linked to cancer adds biological plausibility to our results.

## Introduction

The age-controlled incidence of Ovarian Cancer (OC) is increasing world-wide^1^. While OC is the second most common gynecologic cancer it remains the most lethal. A primary cause of the lethality is the late stage of detection, due to the absence or non-specific nature of its symptoms. Early-stage disease, confined to the ovary has a 5-year survival of 90% while with distant dissemination, this plummets to 29.2% survival^2^. Unfortunately, only 15% of OC cases are diagnosed at the localized stage while 59% have already metastasized at the time of diagnosis^3^.

There is therefore an urgent need to identify accurate screening markers for OC detection. The most widely used biomarker currently is the cancer antigen-125 (CA-125), a membrane glycoprotein. CA-125 has a low sensitivity for early-stage OC. It also suffers from a lack of specificity given that both benign and malignant non-OC disorders can result in elevated serum levels, limiting its utility as a screening agent^4^. Imaging studies on the other hand face challenges in resolving cancers of small size and differentiating malignant and non-cancerous lesions^5^.

There has been a surge in scientific interest in the capture and analysis of circulating tumor cells or cell free nucleic acids (“liquid biopsy”) given its potential for minimally invasive cancer detection. Although the various mechanisms of cell free tumor DNA (cfTDNA) release remain to be definitively established, cfTDNA is released with cell necrosis, apoptosis^6^ along with ongoing release from intact cancer cells. Higher levels of circulating cell free DNA (cfDNA) occur in cancer patients, most of which originate from the neoplastic cells.

DNA methylation generally refers to the addition of a carbon atom (aka “methyl group”) to the cytosine nucleotide in DNA. The methylation of the cytosine in the dinucleotide cytosine-phosphate-guanosine (‘CpG’) remains the most studied epigenetic mechanism. Changes in cytosine methylation is known to alter the 3-dimensional structure DNA and the binding of transcription factors and is thus associated with altered gene expression. This effect is most pronounced for cytosines located in the gene promotor region^7^. Promotor methylation classically results in inactivation of tumor suppressor genes and has been found to be an early event in the ultimate development of ovarian cancer^8^. Further, DNA methylation patterns are reportedly significantly different between OC patients and those with benign ovarian mass or healthy controls^9^. DNA methylation changes are known to occur in multiple locations throughout the genome and thus have lower detection limits compared to mutation or protein marker assays. Indeed, relevant methylation changes that occur in cancer tend to be rare in normal cells^10^. Quantitation of circulating cfTDNA has been evaluated as a diagnostic approach for OC, however systematic reviews suggest the superiority of epigenetic markers over circulating cfTDNA quantitation^11^. For all the reasons outlined above, DNA methylation analysis of circulating cfTDNA hold significant promise for minimally invasive OC detection.

Artificial intelligence is a broad term that refers to the capability of computers to execute tasks that were previously regarded as having an intellectual basis e.g., reason and learning, and therefore the exclusive domain of humans. Machine learning (ML) is a branch of AI where computers ‘learn’ from previous exposure (input data) and based on that ‘knowledge’ can execute functions such as group classification from a new data set. Studies have reported ML to be superior to conventional statistical approaches for group classification in clinical medicine e.g., accurately differentiating cases from unaffected controls^12,13^. Its extraordinary capacity for handling high dimensional or big data makes AI attractive for use in omics studies. The authors have focused on combining AI and epigenomics for minimal invasive disease detection^14,15^. Given the enormous currently untapped potential of Artificial Intelligence in the medical sciences, the current enthusiasm for the use of AI in cancer research^16^ appears warranted.

A limitation of current DNA methylation analysis of cfTDNA in ovarian cancer is the focus on single or a small number of target genes previously identified to be involved in cancer pathogenesis^10^ which limits diagnostic precision. In this preliminary study, we performed genome wide methylation analysis of cfDNA for the minimally invasive detection of OC. A significant objective of Precision Oncology is to elucidate the pathogenesis of cancers with the ultimate intent of developing targeted therapeutics^17,18^. We therefore also evaluated the molecular pathogenesis including gene pathways associated with epithelial OC development.

## Methods

### Study design and methylation profiling

Circulating cfDNA (cFDNA) was profiled using Illumina Infinium MethylationEPIC BeadChip arrays (Illumina, San Diego, CA). In this prospective case–control study, we compared the methylation profile of OC patients with matched age grouped controls. The study was approved by the Institutional Review Board of Beaumont Health System, Royal Oak, MI, USA (IRB#2018-306) and performed in accordance with the Declaration of Helsinki. All participants provided written informed consent. CfDNA was extracted using the blood from 5 OC female cases and 12 female controls. Blood samples were collected in Streck Cell-Free DNA BCT^®^ tubes^19^. Samples were deidentified and processed within 24 h of sample collection. For the primary processing, these were centrifuged at 3000×*g* for 15 min and the plasma was aliquoted into cryogenic vials. The aliquoted plasma samples were stored at − 80 °C until further process. Using QIAamp circulating nucleic acid kit (Qiagen Cat # 55114) the ccfDNA was extracted. All samples were bisulfite converted using EZ DNA Methylation Kit (Zymo, USA) per the manufacturer’s standardized method. Illumina Infinium MethylationEPIC BeadChip arrays (~ 850,000 CpG loci genome-wide) was used for methylation analysis based on manufacturer’s standardized protocol^20^.

### Data pre-processing and statistical analysis

Data analysis was performed using R software version 4.1.1. Raw EPIC array data was processed using minfi package and beta values were normalized using the “noob” normalization method. Probes with detection p-value more than 0.01 were marked as missing. Probes with more than 50% of missing values across all samples were removed. Sex chromosome probes were also removed. Probes with the variance below the first percentile were removed. K nearest neighbor imputation implemented in package *impute* was used to impute missing values. Outliers were detected based on (1) proportion of failed probes with more with 2*sd deviation from the mean considered to be outlier, (2) median probe intensity of unmethylated and methylated probes were computed and samples towards the lower-left most corner of the plot were considered as outliers, (3) Samples with discordant predicted sex (median signal of X and Y chromosomes) and clinically recorded sex were considered outliers, (4) Principal component analysis of centered and scaled beta value matrix was computed and samples deviating more than 2 SDs from the mean of any of the first three principal components were marked as outliers. Blood cell type deconvolution was performed to reduce the variance inflation.

To establish differentially methylated cytosines, robust linear regression modeling was performed after methylation beta values were converted into “M” values using the *limma* package. Condition, age, and cellular proportion estimates of CD8T, natural killer, B cell and monocytes were used as covariates of regression models. The cellular proportion estimated were obtained using *minfi* package. In addition, Removing unwanted variation (RUV) was used to estimate effects of unknown and unwanted variation in the data^21^. Obtained p values were adjusted for multiple testing using false discovery rate correction. EPIC array probe coordinates and R package annotatr were used to associate each probe with CpG island, shore or shelf as well as its relation to genic elements—promoters, intronic, exonic regions, etc. The enrichment of differentially modified cytosines within each region type was tested using Fisher’s exact test.

Pathway enrichment analysis was performed by assigning each EPIC array probe to a UCSC reference gene symbol according to annotation file provided by Illumina. For each gene the most significant methylation locus was retained. The genes were then ranked log p value of the probe multiplied by the sign of fold change. Therefore, hyper-methylated genes were at the top of the ranking and hypo-methylated genes were at the bottom. Gene Set Enrichment Analysis (GSEA)^22^ for WikiPathways was run against a pre-ranked gene list using R packages *fgsea* and *clusterProfiler*. False discovery rate correction was used to correct for multiple testing.

### Logistic regression models

The top 2000 and 5000 CpG loci identified based statistically significant adjusted p-values changes from the promoter regions across the genome were subjected to the logistic regression classifier using Python’s scikit-learn library after pareto scaling and log transformation. The analysis was performed with a stepwise variable selection to optimize all the model components. A k-fold cross-validation (CV) technique ensures the validity of a logistic regression model by randomly dividing the entire sample data into k equal sized subsets, of which only one was used as the validation data for the model, and the remaining subsets are used as training sets. Optimal and robust predictive algorithms were generated.

### Artificial intelligence (AI) analysis

The normalized beta values of overall CpG markers for the intragenic region of human genome was considered to test the performance of predictive models based on AI analysis. We performed the analysis using six predictive algorithms such as, Random Forest (RF), Support Vector Machine (SVM), Linear Discriminant Analysis (LDA), Prediction Analysis for Microarrays (PAM), Generalized Linear Model (GLM) and Deep Learning (DL). Tenfold cross validation and bootstrapping was performed for each model and the average area under the receiver operator characteristics curve (AUC) and 95% confidence intervals, sensitivity, specificity was calculated for the top 5 and 25 markers. The detailed methods on building models were previously described in our earlier publications^15,23^ and provided in the Supplemental Methods section.

### Minimizing overfitting of the models

Overfitting is a risk when small sample sizes are used. To minimize overfitting in the DL model, we used three regularization parameters: L1, which increases model stability and causes many weights to become 0 and L2, which prevents weights enlargement. In addition, to further minimize overfitting in the DL model we used the ‘input dropout ratio’ which controls the amount of input layer neurons that are randomly dropped (set to zero) and controls overfitting with respect to the input data, useful for high-dimensional noisy data^24^. For the other AI models, several parameters were used to tune the models and to overcome the overfitting problem. These were ‘Number of trees’ for RF, ‘classification cost’ for SVM, and ‘threshold amount’ for shrinking toward the centroid for PAM.

## Results

The final study consisted of 5 OC subjects and 12 control subjects. The cohort characteristics and clinical/demographic details are provided in the Supplemental Table 1. The mean (SD) of the age and BMI are not statistically different between the study groups. Histological characteristics and disease stage was provided for the OC cases. Four of the study cases had family history of non-ovarian cancers.

Based on initial quality check, two of the original control samples were found to be PCA outliers and had greater than 50% missing values thus they were removed from further analysis (Supplemental Fig. 1). The cell type deconvolution of cfDNA methylation profiles was performed and whole blood cellular abundance estimates were obtained. The variance inflation analysis revealed that Granulocyte and CD4T abundances were correlated with other covariates and were not included in subsequent linear modeling (Supplemental Fig. 2). Robust linear regression models were fitted to cytosines to identify differentially methylated CpG loci. The models were adjusted for sample age and CD8T, NK, B-cell, Mono cell estimates. Overall, 790,344 probes that passed the quality check were used for logistic regression. The robust linear modeling identified 179,238 differentially modified OC cfDNA cytosines encompassing 23,760 coding genes with the FDR adjusted p-value < 0.05 (Supplemental Table 2). Of these, 90,335 were hypermethylated and 88,903 hypomethylated CpGs. 128,215 CpGs among 179,238 were intragenic (region within gene/promoters/UTRs) CpGs.

### Differentially methylated gene pathways

**Figure 1:**
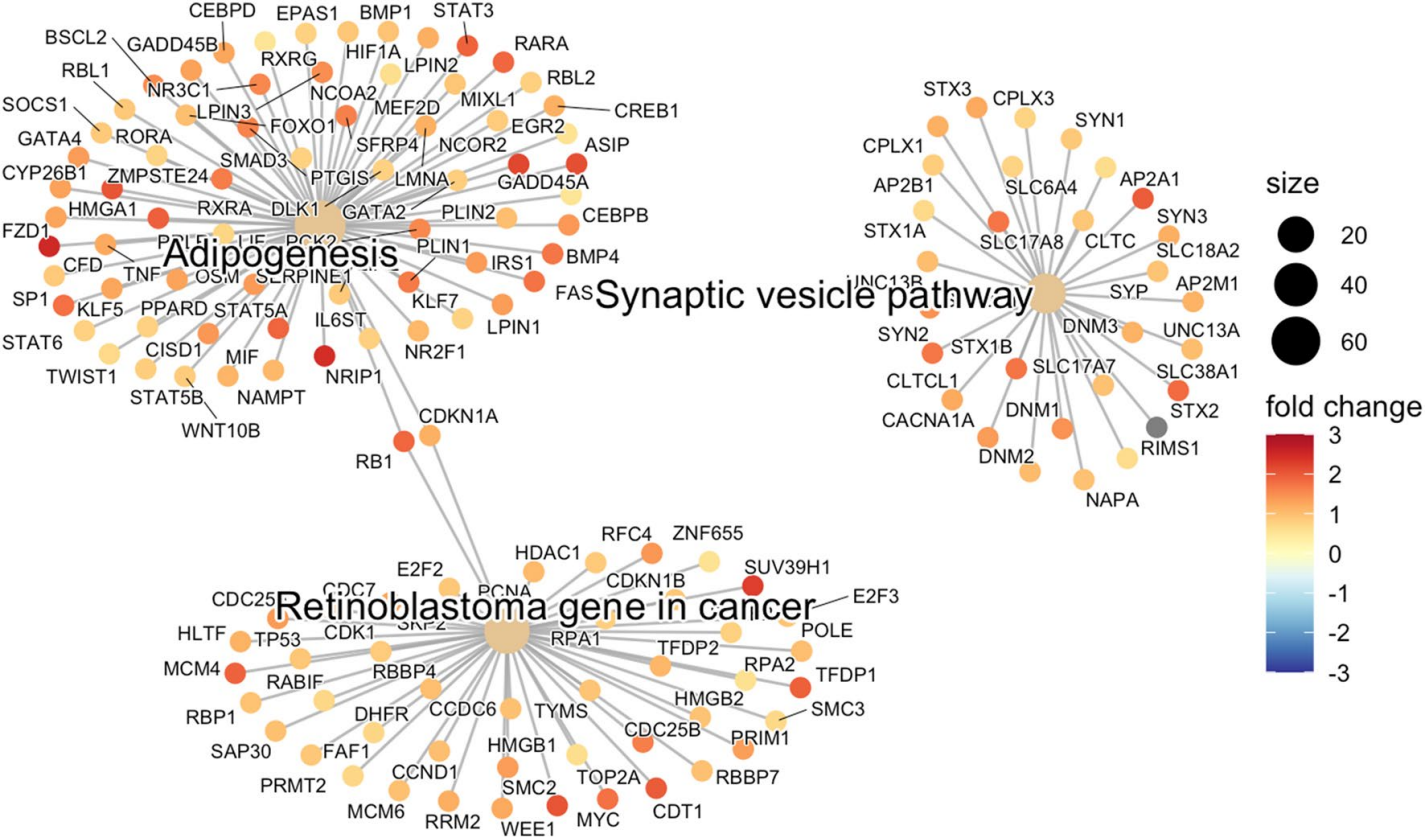
Visual representation of top 3 significantly altered pathways and their constituent genes identified in ovarian cancer circulating cfDNA.

GSEA analysis was performed using all 790,344 probes that had a gene assigned to them. The GSEA WikiPathways identified Adipogenesis (adjusted p-value = 0.00023), Synaptic vesicle pathway (adjusted p-value = 0.0030) and Retinoblastoma gene in cancer (adjusted p-value = 0.0042) as the top 3 pathways. The significant genes are represented as nodes and depicted on Fig. 1. Further statistical and gene details are provided in Supplemental Table 3.

### Logistic regression analysis

**Figure 2:**
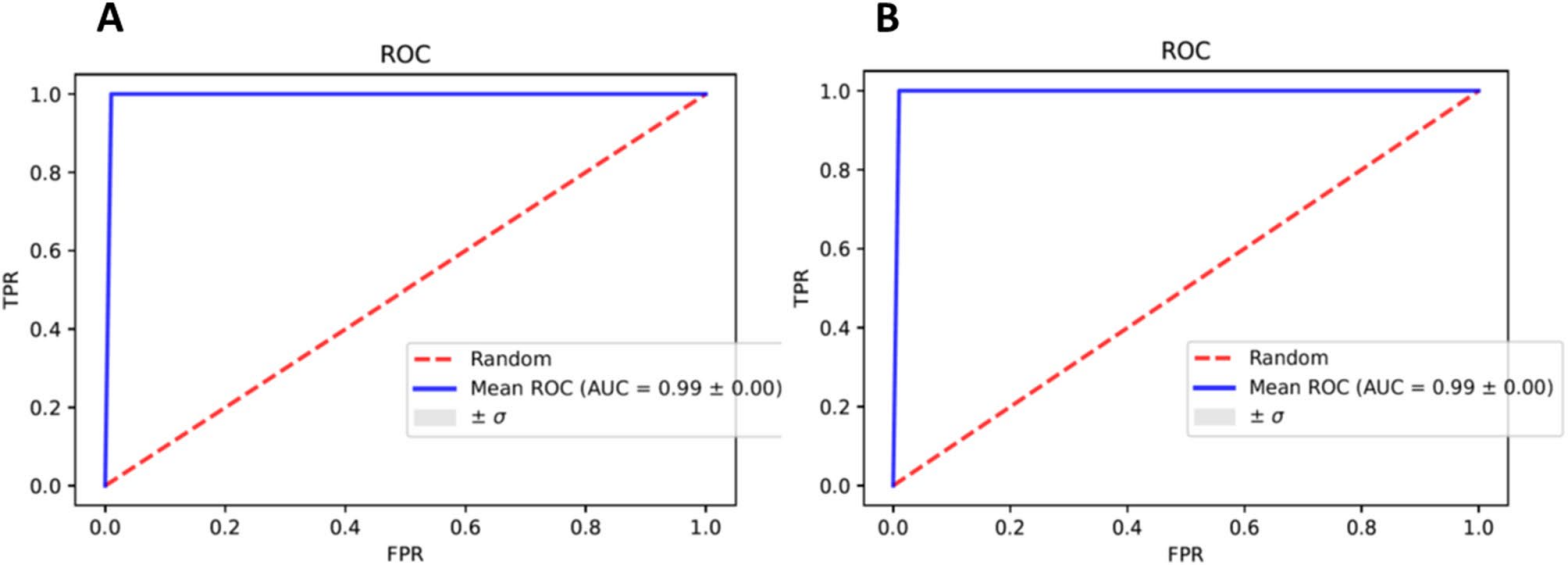
ROC curves provided by analyses of the top CpGs of promoter region- based on individual ability to detect OC (**A**) Top 5000 CpGs, and (**B**) top 2000 CpGs. *TPR* true positive rate, *FPR* false positive rate.

Table 1 lists the results of the five-fold cross validated logistic regression models based on selected promoter CpGs. For the standard logistic regression analysis, we used a Recursive Feature Elimination (RFE) method to find the most discriminative features. Analyzing the top 5000 CpGs a 4-marker predictive model [cg09533869 (*PGCP*), cg16474696 (*MRI1*), cg04888234 (*FCRLA*) and cg08684066 (*HERC6*)] achieved an AUC = 0.99 ± 0.00, sensitivity = 94% and specificity = 98.6%. A similar performance was achieved when analysis was limited to the top 2,000 CpG’s [cg04888234 (*FCRLA*), cg16474696 (*MRI1*), cg17573813 (*FHIT*), cg03299654 (*CD86*)]: AUC = 0.99 ± 0.00, sensitivity = 95% and specificity = 100.0%. Area under the receiver operating characteristic curve (AUROC) for those models therefore reached 99% (Fig. 2).

**Table 1:** Performance evaluation metrics for each logistic regression model to include sensitivity, specificity, accuracy and AUC for top 5000 and top 2000 CpGs in promoter (top four CpG sites selected by RFE^#^ for model building).

Data set has been utilized	Features (CpG sites) selected by RFE^#^	Sensitivity	Specificity	Accuracy	AUC
Top* 5000 CpG in promoter region	cg09533869, cg16474696, cg04888234, cg08684066	94.0	98.6	95.0	99.0
Top* 2000 CpG in promoter region	cg04888234, cg16474696, cg17573813, cg03299654	95.0	100.0	97.0	99.0

Top*—CpGs showing statistically the most significant change between OC cases and control subjects by p-values.

^#^Recursive Feature Elimination (RFE).

### Ovarian cancer detection using artificial intelligence models

Overall intragenic (coding region) CpGs were considered for the AI analysis including DL (Table 2). The performance of top 25 CpG based algorithm using bootstrapping achieved an AUC (95% CI) 1.00 (0.9000–1.0) for three models: DL, RF and SVM, and the other 3 models GLM, PAM and LDA achieved an AUC (95% CI) 0.99 (0.9000–1.0). All 6 models performed with a sensitivity of 100%, with specificity values of 72–88%. For DL the sensitivity was 100% for a specificity of 88%. The detailed result showing the top 25 predictors used in each AI platform are provided on Table 2. Interestingly, CpGs (gene) cg22563825 (*ANO2*), cg16474643 (*ATP11A*), cg05476766 (*AGAP1*), cg20675030 (*ARFGEF2*), cg04388790 (*BBS9*) were consistently ranked as top predictive markers for OC in four of the six AI algorithms evaluated. Using cross validation analysis of CpGs, the top 25 intragenic CpG markers performed with an AUC (95% CI) 1.00 (0.9–1.0) with the DL and SVM models. The other four models (GLM, PAM, RF and LDA) achieved an AUC (95% CI) 0.99 (0.85–1.0) with a sensitivity of 100% in each of the six models and specificity range of 72–88%. The detailed results for algorithms in which CV analysis of the data was performed is shown in Table 3. Finally, as previously mentioned, CpG methylation changes in the promoter regions are more strongly associated with changes in gene expression. We therefore looked at the performance of only CpGs located in the promoter regions (TSS1500, TSS200 and 5′UTR) to see whether they improved OC prediction. We also increased the number of predictive markers per algorithm to 100 to see whether it improved performance. Using bootstrapping, DL achieved an AUC (95% CI) of 1.00 (0.95–1) with sensitivity of 99% and specificity of 81% Supplemental Table 4. Comparable predictive performance was achieved for the different AI platforms when considering only the promoter regions.

**Table 2:** Ovarian cancer detection using circulating cfDNA: artificial intelligence with bootstrapping (top 25 variables).

	SVM	GLM	PAM	RF	LDA	DL
AUC 95% CI	1.0000 (0.9000–1)	0.9992 (0.9000–1)	0.9989 (0.8500–1)	1.0000 (0.9000–1)	0.9997 (0.9000–1)	1.0000 (0.9500–1)
Sensitivity	1.0000	1.0000	1.0000	1.0000	1.0000	1.0000
Specificity	0.7200	0.8100	0.8000	0.7700	0.7500	0.8800

Support Vector Machine (SVM), Generalized Linear Model (GLM), Prediction Analysis for Microarrays (PAM), Random Forest (RF), Linear Discriminant Analysis (LDA) and Deep Learning (DL).

Important predictors in order:

**SVM:** cg22563825, cg16474643, cg05476766, cg20675030, cg04388790, cg02028146, cg11589832, cg21842691, cg24674098, cg05071812, cg04973837, cg13670531, cg23169883, cg17569522, cg08758198, cg03996001, cg16847766, cg16283012, cg01885356, cg17336368, cg02267288, cg14418948, cg23130075, cg12604184, cg18093771.

**GLM:** cg10818896, cg26792783, cg13782386, cg03299654, cg08974492, cg10745724, cg08346273, cg07630078, cg12126686, cg00502292, cg22089454, cg08842907, cg20067526, cg17539962, cg10333415, cg19296509, cg00926889, cg18382353, cg13758494, cg09966430, cg21607700, cg01285783, cg18944099, cg04352288, cg18458597.

**PAM:** cg13670531, cg23169883, cg17569522, cg08758198, cg03996001, cg16847766, cg16283012, cg01885356, cg17336368, cg02267288, cg14418948, cg23130075, cg12604184, cg18093771, cg10333415, cg19296509, cg00926889, cg18382353, cg13758494, cg09966430, cg21607700, cg01285783, cg18944099, cg04352288, cg18458597.

**RF:** cg22563825, cg16474643, cg05476766, cg20675030, cg04388790, cg10818896, cg26792783, cg13782386, cg03299654, cg08974492, cg02028146, cg11589832, cg21842691, cg24674098, cg05071812, cg10745724, cg08346273, cg07630078, cg12126686, cg00502292, cg02267288, cg14418948, cg23130075, cg12604184, cg18093771.

**LDA:** cg05071812, cg04973837, cg13670531, cg23169883, cg17569522, cg08758198, cg03996001, cg16847766, cg16283012, cg01885356, cg22089454, cg08842907, cg20067526, cg17539962, cg10333415, cg19296509, cg00926889, cg18382353, cg13758494, cg09966430, cg22563825, cg16474643, cg05476766, cg20675030, cg04388790.

**DL:** cg22563825, cg16474643, cg05476766, cg20675030, cg04388790, cg02028146, cg11589832, cg21842691, cg24674098, cg05071812, cg10818896, cg26792783, cg13782386, cg03299654, cg08974492, cg10745724, cg08346273, cg07630078, cg12126686, cg00502292, cg02267288, cg14418948, cg23130075, cg12604184, cg18093771.

**Table 3:** Ovarian cancer prediction based on cell free DNA: Artificial intelligence with cross validation (top 25 variables).

	SVM	GLM	PAM	RF	LDA	DL
AUC 95% CI	1.0000 (0.9000–1)	0.9992 (0.9000–1)	0.9989 (0.8500–1)	0.9988 (0.9000–1)	0.9994 (0.9000–1)	1.0000 (0.9500–1)
Sensitivity	1.0000	1.0000	1.0000	1.0000	1.0000	1.0000
Specificity	0.7000	0.8000	0.8100	0.7500	0.7500	0.8800

Support Vector Machine (SVM), Generalized Linear Model (GLM), Prediction Analysis for Microarrays (PAM), Random Forest (RF), Linear Discriminant Analysis (LDA) and Deep Learning (DL).

Important predictors in order:

**SVM:** cg22563825, cg16474643, cg05476766, cg20675030, cg04388790, cg02028146, cg11589832, cg21842691, cg24674098, cg05071812, cg04973837, cg13670531, cg23169883, cg17569522, cg08758198, cg03996001, cg16847766, cg16283012, cg01885356, cg17336368, cg02267288, cg14418948, cg23130075, cg12604184, cg18093771.

**GLM:** cg10818896, cg26792783, cg13782386, cg03299654, cg08974492, cg10745724, cg08346273, cg07630078, cg12126686, cg00502292, cg22089454, cg08842907, cg20067526, cg17539962, cg10333415, cg19296509, cg00926889, cg18382353, cg13758494, cg09966430, cg21607700, cg01285783, cg18944099, cg04352288, cg18458597.

**PAM:** cg13670531, cg23169883, cg17569522, cg08758198, cg03996001, cg16847766, cg16283012, cg01885356, cg17336368, cg02267288, cg14418948, cg23130075, cg12604184, cg18093771, cg10333415, cg19296509, cg00926889, cg18382353, cg13758494, cg09966430, cg21607700, cg01285783, cg18944099, cg04352288, cg18458597.

**RF:** cg22563825, cg16474643, cg05476766, cg20675030, cg04388790, cg10818896, cg26792783, cg13782386, cg03299654, cg08974492, cg02028146, cg11589832, cg21842691, cg24674098, cg05071812, cg10745724, cg08346273, cg07630078, cg12126686, cg00502292, cg02267288, cg14418948, cg23130075, cg12604184, cg18093771.

**LDA:** cg05071812, cg04973837, cg13670531, cg23169883, cg17569522, cg08758198, cg03996001, cg16847766, cg16283012, cg01885356, cg22089454, cg08842907, cg20067526, cg17539962, cg10333415, cg19296509, cg00926889, cg18382353, cg13758494, cg09966430, cg22563825, cg16474643, cg05476766, cg20675030, cg04388790.

**DL:** cg22563825, cg16474643, cg05476766, cg20675030, cg04388790, cg02028146, cg11589832, cg21842691, cg24674098, cg05071812, cg10818896, cg26792783, cg13782386, cg03299654, cg08974492, cg10745724, cg08346273, cg07630078, cg12126686, cg00502292, cg02267288, cg14418948, cg23130075, cg12604184, cg18093771.

## Discussion

Precision Medicine (PM) is a developing field which integrates multimodal data e.g. individual clinical, laboratory and imaging data and most recently ‘omics’ to improve the diagnosis, understanding and treatment of disease^25^. The recent leap in progress in computer sciences means that we now have the tools such as Artificial Intelligence (AI) with which to analyze the torrent of data generated in multi-omics analyses. The impact of AI in PM and cancer continues to grow with applications that include detection, classification, drug development and prognostication^16^. The use of liquid biopsy for OC analysis is a promising area. In OC, circulating tumor cells and tumor DNA have been used preliminarily for OC diagnosis^26^. Multigene mutation analysis of circulating cfDNA drawn prior to surgery was shown to have high diagnostic accuracy^27^. Similar, results were obtained with mutation analysis of circulating cfDNA for the detection of early-stage OC detection^28^.

Methylation patterns are easier to detect than mutations as they are binary signals. Methylation assays have a low limit of detection, e.g., for methylation specific PCR this has been estimated to be as low as 0.01%^29^ and is thus effective for analyzing low levels of circulating tumor DNA. Methylation patterns in single genes such as *RASSF1A* and *OPCML* appear to be effective markers for OC detection^30^. The focus on a single or a few methylation sites has however limited the sensitivity and specificity of this approach for OC detection^31^. As a consequence, we simultaneously evaluated large numbers of potential CpG markers across the genome. Given the small sample size we also used conventional logistic regression analysis for comparison. Parsimonious prediction models using a small number of markers, i.e., four CpGs per model, achieved high AUCs (0.99 ± 0.00), sensitivities and specificities.

AI application in OC was initially focused on integrating data from imaging, histology, clinical and laboratory markers to improve diagnosis, surgery, treatment and prognostication^32^. Here, AI combined with multiple different combinations of CpG markers, statistical and bioinformatic approaches yielded high diagnostic accuracy for OC detection with an AUC close to or equal to 1.0. Despite the small number of cases in the study statistical significance was achieved with the lower confidence interval for AUC ≥ 0.85. This was true when using a small or large number of CpG methylation markers. The spectacular advances of DL in imaging and face recognition have fueled interest in cancer applications such as interpreting radiological images and histologic slides. In our analyses, DL appeared consistently superior to the other AI platforms for OC detection with AUC (95% CI) 1.0 (0.95–1.0), Tables 2, 3 and Supplemental Table 4. Promoter methylation was found to be an early event in OC pathogenesis^8^. We therefore evaluated the utility of CpG methylation confined to the promoter region of thousands of genes across the genome. Comparably high diagnostic performances were achieved with AI algorithms. Overall, the AUC (95% CI) 1.0 (0.80–1.0) and for DL this was AUC (95% CI) 1.0 (0.95–1.0) (Supplemental Table 4). This suggests the potential of promotor methylation analysis using cfDNA for the early detection of OC.

We used AI to rank and thus identify the best predictive markers across different platforms. Five CpGs (genes) were found to be consistently among the top markers in four of the six AI platforms. Based on this approach, the *ANO2* (*TMEM16B*) gene appeared to be the most significant gene among them. This gene functions as Ca^2+^ activated Cl^−^ channels (CaCCs), an ion channel involved in transepithelial ion transport and smooth muscle contraction^33^. Of note, calcium channels play a significant role in controlling cell cycle and resistance to apoptosis^34^. The specific significance of ANO2 relative to OC needs to be investigated. The cg16474643 (*ATP11A)* was the next most significant methylation marker (gene). The (*ATP11A)* gene affects the epithelial to mesenchymal transition (EMT) mechanism observed in pancreatic cancer^35^. During the EMT process, the epithelial cells lose their polarity and are transformed into mesenchymal cells with amplified migratory capacity required in cancer cells^36^. We were unable to find similar information on the impact of this gene in OC. cg05476766 (*AGAP1*) was another significant marker. Single nucleotide polymorphism of *AGAP1* is associated with reduced survival rates in OC patients undergoing treatment with bevacizumab with potential utility in OC detection^37^.

With regards to the cg20675030 (*ARFGEF2*) locus, the gene makes a protein involved in the trafficking of vesicles within the cells. *ARFGEF2* protein activates an ADP-ribosylation factor (ARF). ARFs play a key role in intracellular vesicular trafficking^38^. ARFs function as tumor suppressors by activating p53^39^. Finally, the gene *BBS9* (differentially methylated on cg04388790) codes for the protein Parathyroid Hormone Responsive B1 Gene (PTHB1) which is involved in ciliary defects^40^. *PTHB1* gene polymorphism has been associated with premature ovarian failure^41^. Of note, defects in the primary cilium, a sensory organelle on the surface epithelium of the ovary, has been implicated in tumorigenesis possible through its involvement in ciliary signaling controlled by Sonic Hedgehog pathway^42,43^.

Finally, we summarize the results and potential significance of our Pathway Enrichment analysis. Adipogenesis was found to be dysregulated (adjusted p-value = 0.00023). Adipocytes support the growth of tumor cells in OC by transferring lipids to the neoplastic cells, indicating their importance as an energy source^44^. We identified 69 genes in the adipocyte pathway that were significantly differentially methylated in OC.

The Synaptic vesicle pathway was significantly altered with an adjusted p-value = 0.0030. Some of the genes that actively participate in the synaptic vesicle pathway and neurotransmission, have been previously reported to be involved in OC^45,46^. The genes produce Gamma-aminobutyric acid (GABA) in different cancer cells including OC cells resulting in elevated intra-tumoral GABA level^46^. We found significantly altered methylation in CpGs of 29 genes in the pathway OC in our study. The finding needs further investigation to understand the possible mechanism of these genes in causing OC.

Finally, the Retinoblastoma gene in cancer pathway was found to be epigenetically dysregulated in OC (adjusted p-value = 0.0042). Inactivation of specific *RB1* gene has been linked to OC development. In addition other genes in the pathway have been reportedly linked to malignant transformation in OC patients^47^. We found 10 differentially methylated CpGs in the *RB1* gene and 45 other significantly differentially methylated genes in this pathway in our study.

Our study has limitations. It should be considered exploratory given the small number of subjects used. Clearly large validation studies are warranted. Despite this limitation, high OC diagnostic accuracies based on AUC were found using logistic regression-based models and also using multiple different AI platforms and bioinformatic approaches. Multiple strategies were used to minimize overfitting as detailed in "Methods" section (Minimizing overfitting of the models). In addition, we did not evaluate the capabilities of genome-wide methylation changes in distinguishing OC from benign tumors of the ovary. Prior studies have however found significant methylation differences in circulating cfDNA between these two groups^9^. A notable advantage of our study was the fact that specimens were obtained only from patients with no prior radiation, chemotherapy, or surgical therapies. This is important since, chemo and radiation therapy can themselves induce epigenetic changes confounding whether observed OC changes were due to the cancer itself or the therapy.

## Conclusion

We report the use of AI to identify the top predictive epigenetic markers across the genome. Both logistic regression and AI approaches consistently yielded high diagnostic accuracy for minimally invasive OC detection based on cytosine methylation changes in circulating cfDNA. Similar results were obtained for CpG markers confined to the promoter region which is thought to be involved in early neoplastic transformation. This study demonstrates the potential value of Precision Oncology based on the combination of AI and epigenomic analysis for both the accurate detection of and for the elucidation of the pathogenesis of OC. The latter is critical for the development and deployment of novel targeted therapeutics such as CRISPR-based DNA methylation editing^48^. Larger confirmation studies are clearly warranted.

## Supplementary Information

Supplementary Information.

Supplementary Figure 1.

Supplementary Figure 2.

Supplementary Table 1.

Supplementary Table 2.

Supplementary Table 3.

Supplementary Table 4.

## Data Availability

The data that support the findings of this study are available on request from the corresponding author.
