# Supplementary material for: Precision gynecologic oncology: circulating cell free DNA epigenomic analysis, artificial intelligence and the accurate detection of ovarian cancer
Source: Sci Rep. 2022 Nov 3;12:18625. doi: 10.1038/s41598-022-23149-1 (PMC9633647; doi:10.1038/s41598-022-23149-1)
Supplement: Supplementary file 1 — Supplementary Information. [file 41598_2022_23149_MOESM1_ESM.docx]

**Supplemental Methods:**

**Artificial Intelligence (AI) Analysis:**

**Random Forest** (RF) is a supervised classification algorithm which creates a forest of decision trees that are randomly generated. There is a direct correlation between the number of trees in the forest and the accuracy of the results that are generated. The accuracy of the results is increased by increasing the number of trees. RF has several benefits such as being able to work with missing values, being able to overcome the overfitting problem by utilizing sufficient number of decision trees and analysis of categorical values ^1^. **Support Vector Machine** (SVM) is a very robust algorithm used in bioinformatics as it works well with sparse and noisy data. When used for classification, it separates a given set of binary labelled training data with a hyper-plane that is maximally distant from each other (known as ‘the maximal margin hyper-plane’). For cases in which no linear separation is possible, SVM can work in combination with the technique of ‘kernels’, that automatically realizes a non-linear mapping to a feature space. The hyper-plane identified by the SVM in feature space corresponds to a non-linear decision boundary in the input space. **Linear Discriminant Analysis** (LDA) reduces the number of features or predictors needed to accurately classify and discriminate the groups. LDA easily handles the situation in which the within-class frequencies are unequal, and their performances has been examined on randomly generated test data. This method maximizes the ratio of between-class variance to the within-class variance in any particular data set thereby guaranteeing maximal separability. LDA is simple in approach but it still achieves excellent accuracy. The accuracy achieved is similar to that obtained with more complex methods. LDA is based on the identification of a linear combination of variables (predictors) that best separates the two classes (targets) ^2^. Partitioning Around Medoids (PAM) is a statistical technique for class prediction that was based on gene expression data using the nearest shrunken centroids ^3,4^. This method identifies the subsets of genes that best characterize each class. **Generalized Linear Models** (GLMs) are a broad class of models that include linear regression, ANOVA, Poisson regression, log-linear models and others ^3,4^.

**Deep Learning**

Deep Learning (DL) is a form of representation learning that uses multiple transformation steps to create very complex features. DL is categorized into feed-forward artificial neural networks (ANNs), which uses more than one hidden layer (y) that connects the input (x) and output layer (z) via a weight (W) matrix. The weight matrix is expected to minimize the difference between the input and output layers and is considered as the best AI approach.

Deep learning models have multiple hyper-parameters. Finding the best configuration for these parameters in such a high dimensional space is not a trivial challenge. The process of setting the hyper-parameters requires expertise and extensive trial and error. There are no simple or easy methods to set hyper-parameters. The hyper-parameters act as knobs which can be tweaked during the training of the model. For our model to provide the best result, we needed to find the optimal value of these hyper-parameters. We used grid search to determine the best set of parameters. With grid search, we tried every possible configuration of the parameters. We first defined a grid on n dimensions, where each of these maps for a hyper-parameter. For each dimension, we then defined the range of possible values. Search for all the possible configurations and based on the results identify the best one. Finally, the grid search algorithm found the best set of parameters to give us the highest AUC result. The parameters and the ranges that we utilized in the models were as follows:

hyper_params <- list(

activation=c("Rectifier","Tanh"),

hidden=list(c(100),c(200),c(10,10),c(20,20),c(50,50),c(30,30,30),c(25,25,25,25)),

input_dropout_ratio=c(0,0.05,0.1),

hidden_dropout_ratios=c(0.6,0.5,0.6,0.6),

l1=seq(0,1e-4,1e-6),

l2=seq(0,1e-4,1e-6),

train_samples_per_iteration =c(0,-2),

epochs = c(500),

momentum_start=c(0,0.5),

rho=c(0.5,0.99),

quantile_alpha=c(0,1),

huber_alpha=seq(0,1) )

**Modeling & Evaluation:**

Two-step validation was utilized for these analyses. There were two different data sets used in this study. The first was utilized to build the model and test it, the second one was used to independently validate the previously developed model.

While using the two-step validation method, two different techniques were utilized to find out the best model and calculate the performance metrics: 5-fold Cross validation and Bootstrapping.

1. **5-fold Cross Validation:** The data should split into training and testing sets to train the model first with a portion of the data and then test it by using the remaining portion. We preferred to split the data set as 80% training and 20% testing since this ratio is generally used in medium size data sets. The model was fit on the training set, and the fitted model was used to predict the responses for the observations in the hold-out set. Estimates were used to select best model, and to give an idea of the test error of the final chosen model. Idea was to randomly divide the data into 5 equal-sized parts. We left out part 5, fit the model to the other 4 parts (combined), and then obtained predictions for the left-out 5th part. This was done in turn for each part k = 1, 2...5, and then the results were combined. This process was repeated a total of one hundred times and the average AUC, sensitivity, specificity and 95% confidence intervals for the test set were calculated. Then, as the validation step, AUC, sensitivity, specificity and 95% confidence intervals for the validation data set were calculated, too.
2. **Bootstrapping:** The dataset that was utilized in this study was imbalanced in terms of number of cases vs controls. To address the issue of imbalance, bootstrapping technique was utilized. Bootstrapping is a flexible and powerful statistical tool that allows us to use the computer to mimic the process of obtaining new data sets, such that we were able to estimate the variability of our estimate without obtaining additional samples. Rather than repeatedly obtaining independent data sets from the population, we instead obtained distinct data sets by repeatedly sampling observations with replacement from the original data set. Each of these “bootstrap data sets” was created by sampling with replacement and was the same size as our original dataset. As a result, some observations appeared more than once in each bootstrap data set and some not at all. To estimate prediction error using the bootstrap, we used each bootstrap dataset as our training sample, and the original sample as our test sample. This process was repeated a total of ten times and the average AUC, sensitivity, specificity and 95% confidence intervals for the test set were calculated. Then, as the validation step, AUC, sensitivity, specificity and 95% confidence intervals for the validation data set were calculated, too.

**References:**

1 Huang, J. H. *et al.* Using random forest to classify T-cell epitopes based on amino acid properties and molecular features. *Anal Chim Acta* **804**, 70-75, doi:10.1016/j.aca.2013.10.003 (2013).

2 Liland, K. H. Multivariate methods in metabolomics – from pre-processing to dimension reduction and statistical analysis. *TrAC Trends in Analytical Chemistry* **30**, 827-841, doi:<https://doi.org/10.1016/j.trac.2011.02.007> (2011).

3 Alakwaa, F. M., Chaudhary, K. & Garmire, L. X. Deep Learning Accurately Predicts Estrogen Receptor Status in Breast Cancer Metabolomics Data. *J Proteome Res* **17**, 337-347, doi:10.1021/acs.jproteome.7b00595 (2018).

4 Candel, A., Parmar, V., LeDell, E. & Arora, A. *Deep Learning with H2O*. (2018).
