# Supplementary figures and images for: Precision gynecologic oncology: circulating cell free DNA epigenomic analysis, artificial intelligence and the accurate detection of ovarian cancer
Source: Sci Rep. 2022 Nov 3;12:18625. doi: 10.1038/s41598-022-23149-1 (PMC9633647; doi:10.1038/s41598-022-23149-1)

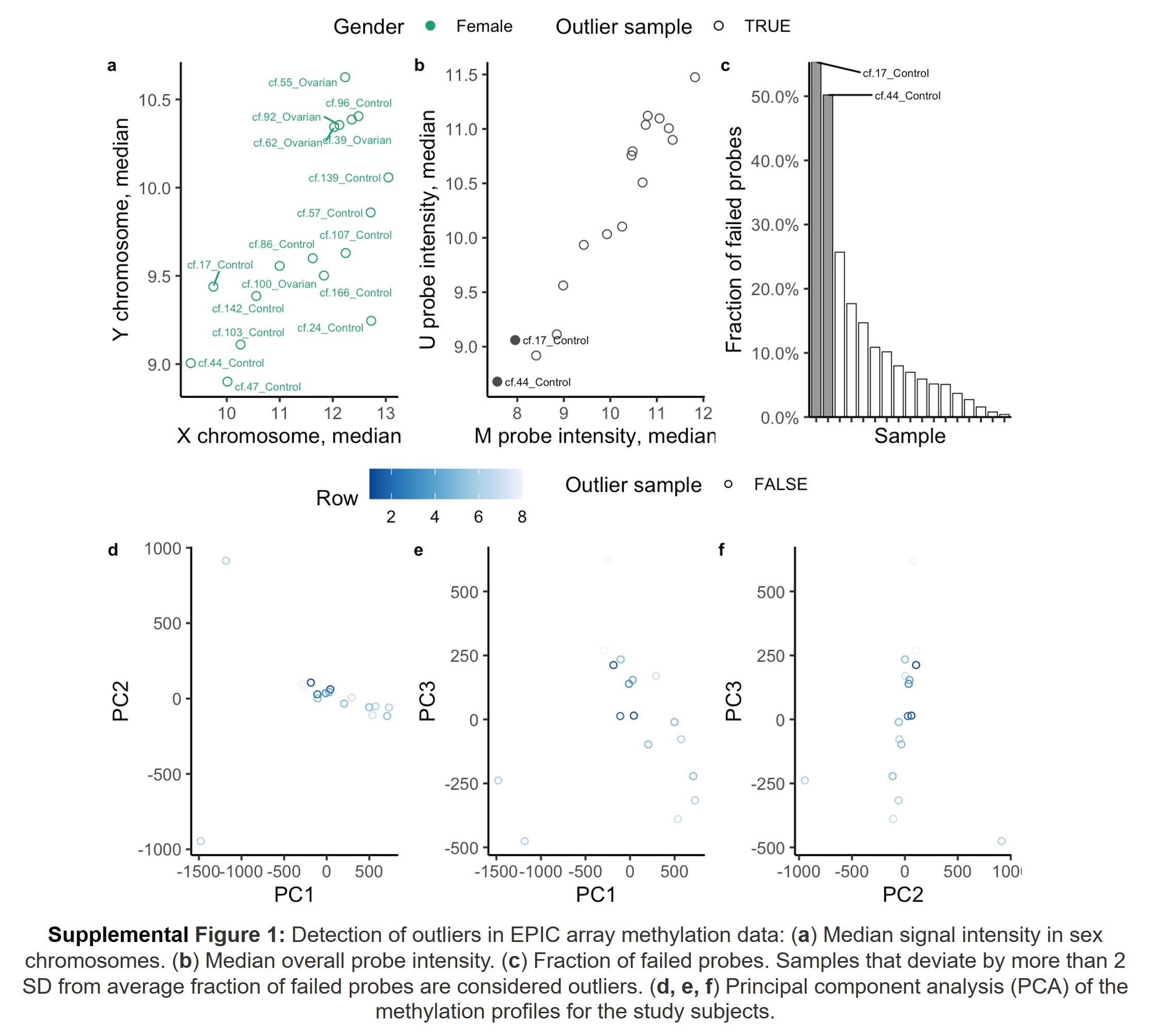

Supplement: Supplementary file 2 — Supplementary Figure 1. [file 41598_2022_23149_MOESM2_ESM.jpg]

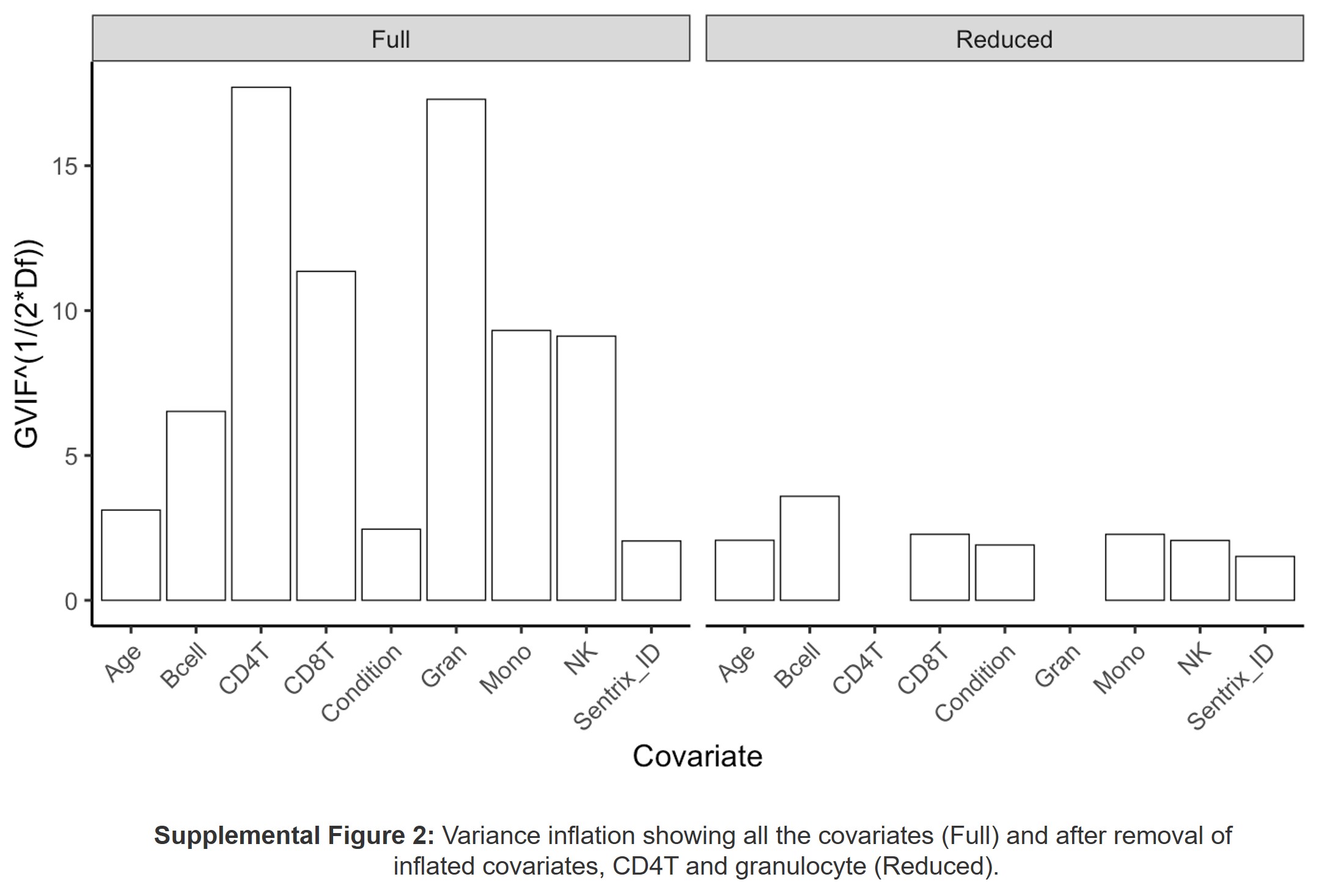

Supplement: Supplementary file 3 — Supplementary Figure 2. [file 41598_2022_23149_MOESM3_ESM.jpg]
