# Supplementary material for: Precision gynecologic oncology: circulating cell free DNA epigenomic analysis, artificial intelligence and the accurate detection of ovarian cancer
Source: Sci Rep. 2022 Nov 3;12:18625. doi: 10.1038/s41598-022-23149-1 (PMC9633647; doi:10.1038/s41598-022-23149-1)
Supplement: Supplementary file 4 — Supplementary Table 1. [file 41598_2022_23149_MOESM4_ESM.docx]

**Supplemental Table 1:** The demographic characteristics (and histology) of ovarian cancer cases and controls.

| **Parameter** | **Cases** | **Controls** | **p-value** |
| --- | --- | --- | --- |
| Number of patients | 5 | 12 | - |
| Race - Caucasian | 5 | 12 | - |
| Gender - Female | 5 | 12 |  |
| Age - Mean (Standard deviation) | 66.2 (18.14) | 67.8 (12.96) | 0.98 (T) |
| BMI - Mean (Standard deviation) | 25.1 (5.26) | 26.9 (6.64) | 0.90 (T) |
| **Histological type and stages of ovarian cancer study subjects** | | | |
| **Histological type** | | | **Stage** |
| High grade serous peritoneal carcinoma | | | IIIA |
| High grade serous carcinoma | | | pT3 N1a |
| High grade serous ovarian cancer (BRCA2 positive) | | | IIIC |
| High grade serous carcinoma, left fallopian tube | | | pT2B, pNX, PMX |
| Mixed serous and endometroid carcinoma high grade ovary | | | pT1c2 NX |

*T – T test
