# Supplementary material for: Precision gynecologic oncology: circulating cell free DNA epigenomic analysis, artificial intelligence and the accurate detection of ovarian cancer
Source: Sci Rep. 2022 Nov 3;12:18625. doi: 10.1038/s41598-022-23149-1 (PMC9633647; doi:10.1038/s41598-022-23149-1)
Supplement: Supplementary file 7 — Supplementary Table 4. [file 41598_2022_23149_MOESM7_ESM.docx]

**Supplemental Table 4:** Ovarian cancer prediction using cell free DNA: Artificial intelligence with bootstrapping based on top 100 variables [ analysis confined to promoter region (TSS1500, TSS200 and 5'UTR).

|  | SVM | GLM | PAM | RF | LDA | DL |
| --- | --- | --- | --- | --- | --- | --- |
| AUC  95% CI | 1.0000  (0.8000-1) | 0.9965  (0.8000-1) | 0.9985  (0.8500-1) | 0.9995  (0.8000-1) | 0.9988  (0.8500-1) | 1.0000  (0.9500-1) |
| Sensitivity | 0.9800 | 0.9800 | 0.9850 | 1.0000 | 0.9800 | 0.9900 |
| Specificity | 0.7200 | 0.7300 | 0.7400 | 0.7300 | 0.7200 | 0.8100 |

Support Vector Machine (SVM), Generalized Linear Model (GLM), Prediction Analysis for Microarrays (PAM), Random Forest (RF), Linear Discriminant Analysis (LDA) and Deep Learning (DL)

Important predictors in order:

**SVM:** cg04888234, cg09017289, cg24721517, cg23130075, cg19178509, cg18011148, cg06350097, cg10604507, cg10432423, cg01885356, cg16084190, cg18944099, cg19296509, cg15247483, cg00659129, cg26948066, cg19811863, cg24509398, cg08974492, cg22089454, cg26502984, cg06836480, cg08497204, cg12101586, cg18799867, cg00368370, cg16985667, cg16847766, cg10625737, cg20922821, cg05301852, cg12349227, cg07170092, cg17844006, cg25536014, cg17521773, cg12667818, cg12943208, cg07766743, cg20130965, cg19144691, cg12682976, cg06329106, cg10256162, cg06625777, cg08044694, cg04226745, cg04550237, cg02955259, cg22360146, cg10833066, cg07761646, cg04778337, cg10503840, cg11441536, cg02995319, cg25982308, cg18443350, cg13559810, cg17509836, cg16871475, cg17190729, cg20403760, cg22049769, cg07861484, cg25575628, cg06982089, cg17897391, cg16166011, cg12921613, cg00617568, cg04133293, cg02297709, cg03075852, cg15269115, cg22238931, cg20438269, cg00431912, cg12969902, cg16716983, cg03378815, cg07399628, cg07208231, cg03257107, cg19921581, cg11684302, cg23812489, cg01459854, cg13271731, cg21481950, cg23562152, cg26534489, cg22361075, cg26446535, cg19015832, cg22013645, cg26036288, cg01488217, cg23378491, cg19518503

**GLM:** cg26623759, cg15116108, cg25880954, cg17841452, cg17503596, cg04790749, cg20912449, cg07488259, cg25750999, cg12458171, cg03401312, cg00186909, cg08672220, cg06814878, cg25913923, cg24494707, cg27221067, cg10066125, cg19495714, cg05258927, cg21894716, cg16437134, cg26160945, cg12337114, cg08198488, cg25152942, cg05797594, cg22921228, cg23710776, cg00080763, cg04613450, cg24516399, cg08605991, cg17071104, cg01552173, cg25153769, cg01010338, cg11979271, cg03673190, cg06975979, cg23196205, cg11005998, cg17827650, cg05868019, cg11638472, cg21206770, cg24813432, cg09490424, cg22908882, cg26256263, cg20847746, cg10186564, cg16556679, cg02438477, cg20272884, cg01160347, cg18135938, cg08725083, cg16599830, cg04721884, cg21616243, cg11050977, cg04851992, cg20343467, cg16179239, cg10487521, cg02897172, cg17040924, cg21592824, cg24142104, cg21503490, cg24571875, cg00961617, cg02118765, cg26594419, cg07384913, cg21609096, cg11182645, cg00647719, cg22790986, cg18200865, cg26588061, cg11293764, cg27443052, cg19772066, cg26827041, cg05342795, cg23618124, cg02478023, cg05005235, cg10123619, cg03829195, cg19692240, cg05572627, cg10999598, cg07157834, cg24870982, cg12218079, cg15138570, cg18913171

**PAM:** cg10759591, cg14459668, cg07945733, cg20264369, cg03113184, cg04971043, cg14972625, cg17645407, cg19899066, cg17539962, cg02413370, cg04622001, cg05207355, cg01709096, cg09363143, cg26792783, cg03299654, cg26987645, cg12126686, cg19696718, cg11254573, cg20479758, cg00021476, cg14166197, cg22720562, cg20214004, cg05016698, cg16289210, cg21216220, cg14975160, cg22800543, cg00290158, cg11222557, cg00822308, cg10701847, cg01333707, cg11678864, cg25407736, cg10168351, cg04191300, cg05193832, cg07887985, cg16536561, cg27472156, cg06091475, cg03240324, cg23984908, cg22334171, cg18213257, cg23752454, cg16604299, cg11906127, cg01423277, cg08907436, cg13185177, cg13959024, cg11857452, cg17220055, cg16748008, cg12570134, cg04114401, cg18995143, cg19442493, cg12408229, cg03538137, cg09621603, cg08233331, cg22105158, cg25633130, cg22590775, cg16234086, cg21181453, cg08408091, cg12219915, cg14105901, cg05215830, cg14509403, cg21697944, cg21377071, cg14667748, cg15337846, cg20479660, cg04087920, cg17170133, cg08177041, cg06908855, cg13732201, cg20772106, cg08714389, cg07228241, cg02339223, cg06858263, cg09584978, cg00658590, cg13392351, cg20168080, cg08116315, cg19820052, cg05723953, cg05361406

**RF:** cg06878786, cg26944725, cg20286956, cg24411778, cg22094019, cg14387182, cg03563562, cg13898013, cg01574823, cg06345247, cg15774752, cg07835283, cg13831540, cg02480685, cg27562023, cg24667736, cg17167021, cg15874981, cg14706519, cg02235529, cg06268905, cg22311616, cg09222505, cg24402990, cg12085265, cg03146575, cg20138860, cg12718519, cg08622098, cg03597644, cg27565556, cg11961495, cg05712873, cg07792708, cg06999043, cg17194270, cg01083121, cg23173240, cg15099537, cg01821656, cg15453644, cg15429655, cg03701870, cg12237926, cg15001728, cg05477521, cg24805342, cg27598576, cg19680908, cg25733174, cg24696810, cg03311522, cg25926001, cg02002240, cg05941155, cg02923856, cg09533869, cg24492508, cg12822338, cg06578786, cg00027619, cg27185512, cg15324197, cg09999451, cg13980623, cg14489199, cg10426163, cg26147314, cg07481154, cg20931294, cg12517109, cg22904102, cg00705730, cg07084627, cg00614959, cg27574547, cg26787863, cg14868265, cg25461308, cg15330738, cg13497866, cg11855354, cg11152012, cg23900606, cg19964040, cg01920980, cg19002413, cg06305026, cg19470726, cg23891364, cg20221381, cg20351149, cg12891476, cg15796926, cg15750696, cg11331516, cg17852507, cg02593766, cg13239126, cg24481177

**LDA:** cg01552173, cg01885356, cg03673190, cg07488259, cg10432423, cg10604507, cg03401312, cg04790749, cg04888234, cg06878786, cg08198488, cg08672220, cg09017289, cg06814878, cg00659129, cg10066125, cg02235529, cg06350097, cg08497204, cg08974492, cg00186909, cg05258927, cg06836480, cg00080763, cg00368370, cg04613450, cg05205432, cg06625777, cg06975979, cg07170092, cg08605991, cg08837884, cg09415148, cg09490424, cg10625737, cg11005998, cg00822308, cg01010338, cg01160347, cg02438477, cg02897172, cg04851992, cg05301852, cg05797594, cg05868019, cg05921500, cg06329106, cg07766743, cg08725083, cg10256162, cg11293764, cg00647719, cg00961617, cg02118765, cg02995319, cg03171708, cg03268293, cg04180085, cg04204557, cg04226745, cg04721884, cg05342795, cg05723953, cg07384913, cg07761646, cg07777909, cg07861484, cg08538258, cg08907436, cg10186564, cg10487521, cg10833066, cg11182645, cg00287012, cg00407962, cg00431912, cg00617568, cg01459854, cg01488217, cg02297709, cg02478023, cg02955259, cg02981828, cg03257107, cg03378815, cg03574115, cg03829195, cg04299266, cg04550237, cg04778337, cg04935965, cg05005235, cg05618517, cg06842329, cg07157834, cg07208231, cg07399628, cg07753223, cg07974303, cg08050114

**DL:** cg18047489, cg12222574, cg11137578, cg16673286, cg21006745, cg18869485, cg03833569, cg12038981, cg25485192, cg10490335, cg26318513, cg27088466, cg24803853, cg23384428, cg09581611, cg22391029, cg22874407, cg26472572, cg19966472, cg24024424, cg14311237, cg10752774, cg23174744, cg25225155, cg21224509, cg19273756, cg09835424, cg08449975, cg17451176, cg16445648, cg17117814, cg20916520, cg24149502, cg04080041, cg02851558, cg03827386, cg16504019, cg09823893, cg08538258, cg09925075, cg05163057, cg11535366, cg10164885, cg18048104, cg26874859, cg03982676, cg19671038, cg13512230, cg02602586, cg25386597, cg02326566, cg18766762, cg08240530, cg27058404, cg25020925, cg14533192, cg19868934, cg12502564, cg21158631, cg20464151, cg11603030, cg22855860, cg03655330, cg05204908, cg27488370, cg19631779, cg25645178, cg17339958, cg02168792, cg11919646, cg21986777, cg23034757, cg23442112, cg10805265, cg11000817, cg02892978, cg16316394, cg26324708, cg01224235, cg21926290, cg11869215, cg16338819, cg18303397, cg05175337, cg20716202, cg23348010, cg10733115, cg15775674, cg27079371, cg11259065, cg24356707, cg22186488, cg18079201, cg20251225, cg16181505, cg12858336, cg25600472, cg00094751, cg05998841, cg26179590
